# Electropolymerized nanoporous polymeric SPME coatings: preparation and characterization by small angle X-ray scattering and scanning electron microscopy

**DOI:** 10.1007/s00706-013-1115-3

**Published:** 2014-01-24

**Authors:** Boguslaw Buszewski, Pawel Olszowy, Stanislaw Pikus, Maciej Kozak

**Affiliations:** 1 Department of Environmental Chemistry and Bioanalytics, Faculty of Chemistry, Nicolaus Copernicus University, Gagarina 7, Torun, Poland; 2 Department of Crystallography, Faculty of Chemistry, Maria Curie-Skłodowska University, Maria Curie-Skłodowska 3, 20-031 Lublin, Poland; 3 Department of Macromolecular Physics, Faculty of Physics, Adam Mickiewicz University, Umultowska 85, 61-614 Poznan, Poland

**Keywords:** Polymerization, Extraction, X-ray scattering, Porosity measurements, Electron microscopy

## Abstract

**Abstract:**

Polymeric polypyrrole and polythiophene solid phase microextraction (SPME) coatings were prepared using electropolymerization with a linear sweep voltammetry technique. Physicochemical properties were measured using different methods, in particular small angle X-ray scattering and scanning electron microscopy. By using innovative approaches for pore size measurement, we were able to calculate a maximum of the pore size range from 80 to 90 nm. Additionally, film thicknesses measured from 90 to 150 μm. Using scanning electron microscopy, we describe the characteristics of polymer growth on the support surface.

**Graphical abstract:**

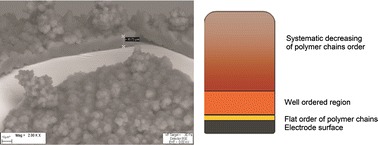

## Introduction

In the past decade, conjugated polymers have found several applications in analytical chemistry as components of chemical sensors (see, for example, [1, 2]) or biosensors [3]. Another analytical application of these polymers should also be pointed out, namely as new materials in solid phase microextraction (SPME) processes. This method, invented by Arthur and Pawliszyn [4, 5] at the end of the last century, was initially focused on polysiloxane and polyacrylate sorbents [6, 7] in environmental and food analysis. Applications of SPME in pharmaceutical analysis forced researchers to develop more porous materials which might be used as sorbents in the extraction. Commercially available sorbent coatings have nonporous surfaces [8], and the extraction efficiencies of these surfaces are usually related to the interactions between the surface and the molecules. In the case of antibiotics, which contain more than one functional group in each molecule, such interactions are not sufficient to obtain reliable results. For this reason and aiming at this application, we have developed a synthesis procedure for porous polymers on a selected conductive (usually metal) surface. These electrochemically deposited coatings of polypyrrole, polythiophene, polyaniline, or their derivatives have been applied in the analysis of medically important drugs from both aqueous solution and human plasma samples [9–12]. Direct (in vivo and pseudo-in vivo) and indirect (in vitro) analyses of extraction coatings have been developed for use in artificial human blood systems [heart–lung machines (HLMs)] [13, 14].

Porosity and pore size distribution measurements are essential for the selection of sorbent materials, especially for matching of the molecule size to appropriate adsorbents. This is why it is important to determine the pore size distribution of organic polymeric sorbents used in SPME. Low-temperature nitrogen adsorption is the method which is commonly used to measure pore area and its size distribution, especially for sorbents based on silica or carbon when the amount of material is sufficient. Unfortunately, in the case of synthetic, electrochemically deposited polymers, application of this procedure is very often impossible. For this reason, we have developed a new method of pore size distribution function determination for electropolymerized conjugated polymer coatings and fibers using small angle X-ray scattering (SAXS).

## Results and discussion

**Fig. 1:**
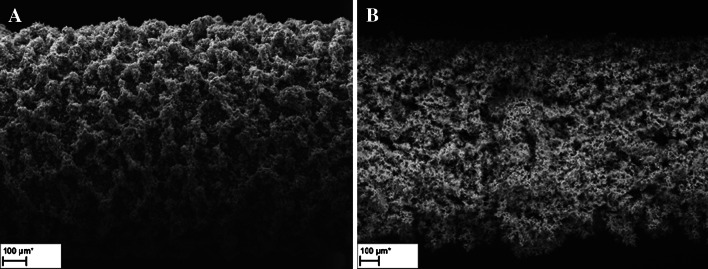
Scanning electron micrographs of polypyrrole (**a**) and polythiophene (**b**) films

Electrochemical polymerization based on linear sweep voltammetry is very useful for preparation of sorbent materials for SPME based on electroactive monomers. Investigations by scanning electron microscopy (SEM) enable measurements of fiber thickness, which ranged from 90 to 95 μm for polypyrrole and from 145 to 155 μm for polythiophene coatings (Fig. 1).

**Fig. 2:**
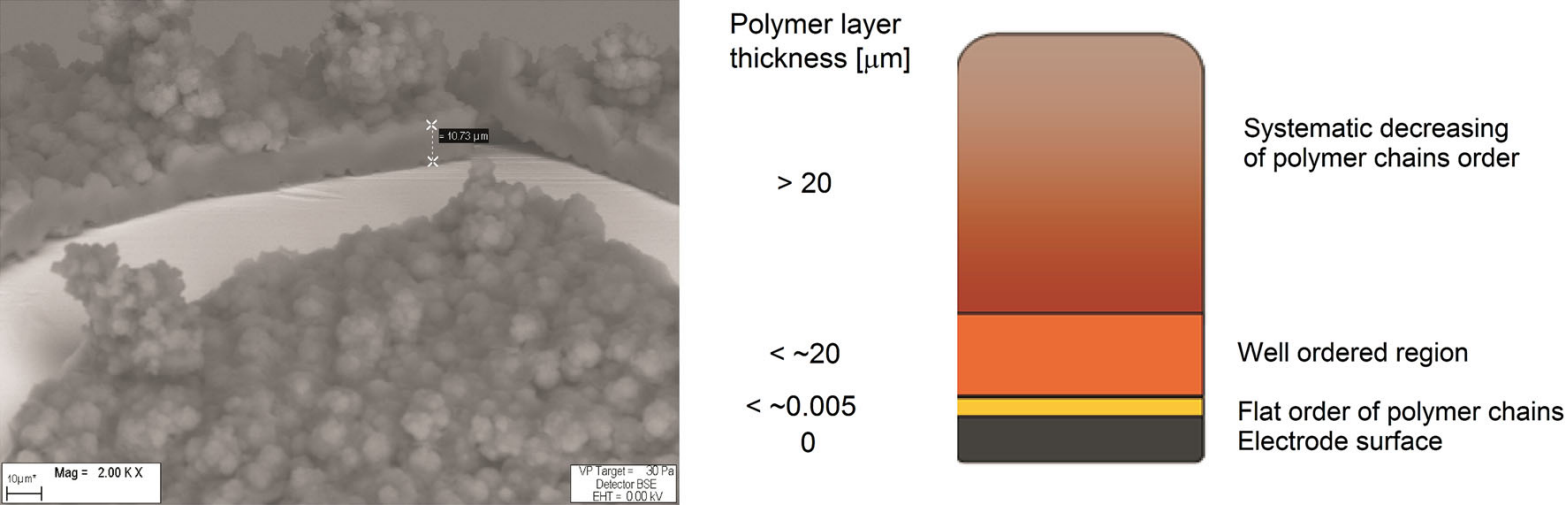
Deposition of polypyrrole coatings on the electrode surface during electropolymerization

The applied electropolymerization method enables observation of the polymer growth on the electrode surface, and we compared our values with those previously described by Tourillon [15] and Roncali [16]. In the first step of polymerization, a thin polymer film grows on the electrode surface (Fig. 2). Polymer chains then coat the metal surface horizontally (monolayer) in a well-ordered manner. This layer has thickness of up to 5 nm and is no higher than the thickness of one polymer chain. Creation of such a film on the surface can be observed by use of near-edge X-ray absorption fine-structure (NEXAFS) spectroscopy. We observed the next layer of film with thickness of 10–12 µm by applying SEM. Other laboratories have observed this layer to be up to 20 μm thick [17]. The magnitude of this region is dependent upon the conditions applied during the polymerization process. Dendrites grow on the well-ordered surface, and in this case fibers with thickness less than 300 μm are desirable. For greater thickness, the polymer structure and properties are more difficult to reproduce. However, when applying identical conditions and apparatus (electrochemical cell and electrodes), it is possible to obtain the same SPME coatings even above the mentioned thickness.

Polymeric coatings, after characterization by SEM, were further subjected to porosity measurements. As mentioned above, in the case of organic polymers, the low-temperature nitrogen adsorption method does not produce useful results due to volatiles remaining after synthesis. Results from the pressure method are marred by errors associated with release of volatiles from polymers during measurements. Hence, an additive effect may be observed and should be taken into consideration when calculating the porosity using this method.

**Fig. 3:**
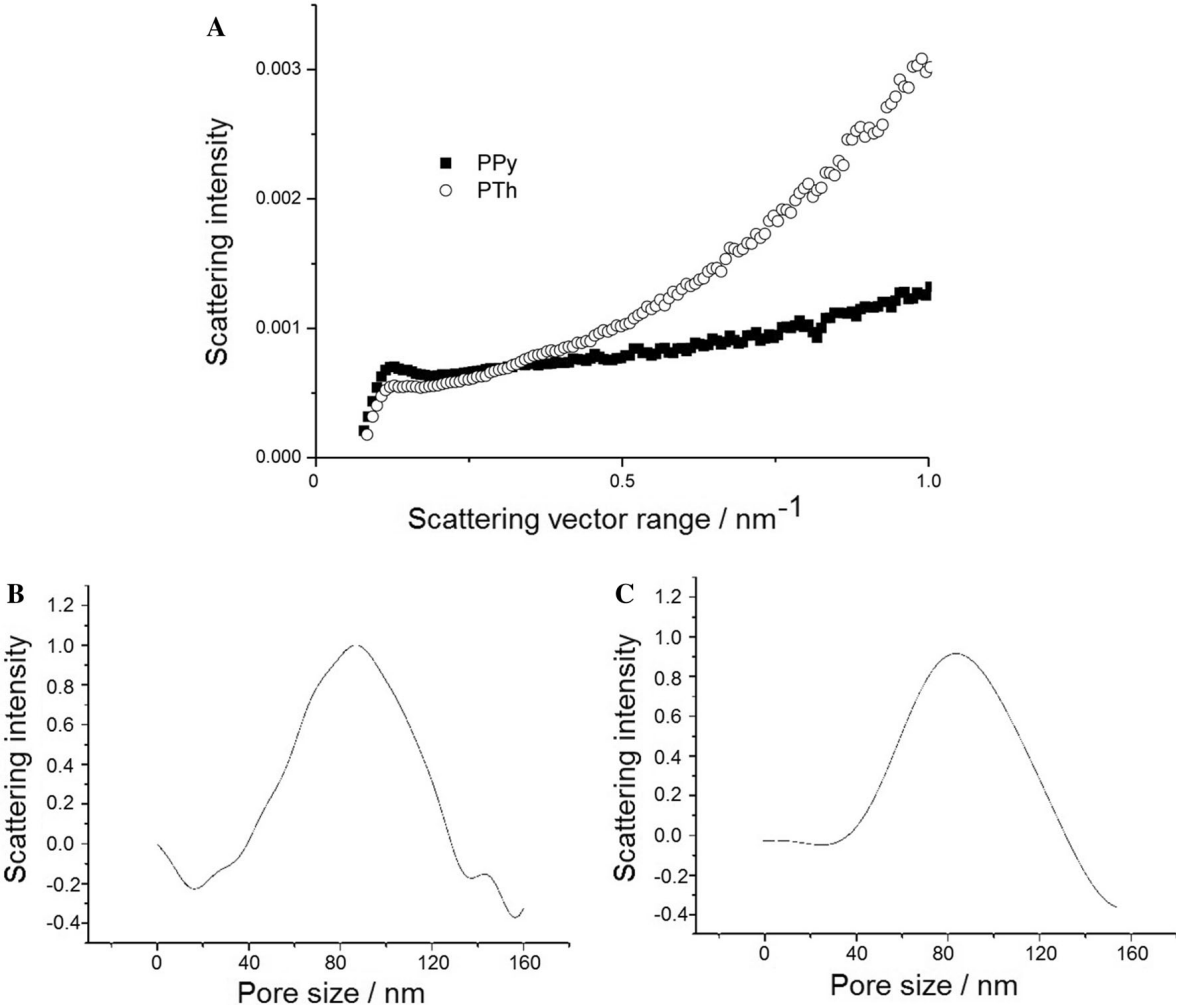
SAXS measurements: Porod plot of SAXS data (**a**), and pore size distribution functions of polypyrrole (**b**) and polythiophene (**c**) coatings

On the other hand, SAXS measurements may be applied for analysis of polymeric porous materials, because the pores and polymeric skeleton can be treated as two phases with different electronic density values. The scattering of X-ray radiation from the tested samples was relatively weak because of the inhomogeneity in the size and shape of pores, being close to the detection limit of the applied method. However, the observations on both samples were almost in accordance with Porod’s law. For the polypyrrole sample, SAXS data showed a sharp rise at the beginning of the Porod plot, then a slight decrease, and later the curve was almost parallel to the *s*-axis. For polythiophene samples, the Porod curve showed a significant increase for high *s* values, suggesting the existence of heterogeneity in the sample. Hence, in this work, the pore size distribution function was calculated using Vonk’s method [18–21] (described in detail in [19] as method II), but the accuracy of the resulting pore size distribution function was not very high. Nevertheless, as mentioned above, other methods did not allow for any measurements of the porosity of the organic polymers. In this regard, the SAXS method is a very good alternative to standard porosimetry. In addition, we have already used the SAXS method for successful characterization of other porous materials (molecular sieves, polymers) [21–24]. Based on the performed measurements and calculations, the pore size distribution ranged from 20 to 160 nm for polypyrrole (max value = 86 nm) and from 40 to 120 nm for polythiophene (max value = 82 nm) (Fig. 3).

For polypyrrole, the pore size range may be narrowed to that of the polythiophene material. The initial and final slopes of the curve for polythiophene are smoother compared with that of polypyrrole. This is especially visible at the beginning of the presented dependence. These fluctuations depend on the formation of the layer during electropolymerization.

In addition to the fact that the studied samples obeyed Porod’s law, it can be concluded that the changes of the electron density at the polymer matrix–pore interface are stepwise and relatively sharp. There is not a gradual change of the electron density, and therefore it cannot be concluded that an intermediate layer exists in terms of changes in the electron density in the pores on the surface.

## Conclusions

We present an approach for describing the pore size distribution of polypyrrole and polythiophene SPME coatings using a SAXS method, which has often been used for characterization of porous nanomaterials. Polypyrrole and polythiophene SPME coatings possess a pore size in the range between 20 to 160 nm and 40 to 120 nm, respectively. Use of SEM allowed evaluation of the mechanism of film development on the electrode surface. On the basis of these measurements, it was shown that a very well-ordered region is formed on the support at the beginning, after which dendrite structures form.

## Experimental

### Preparation of SPME coatings

Electropolymerization was performed using a homemade electrochemical cell coupled with a high performance potentiostat–galvanostat (PGSTAT128N series; Metrohm-Autolab B.V., Utrecht, The Netherlands). All chemicals and reagents were of high performance liquid chromatography (HPLC) or analytical grade. Acetonitrile was purchased from Mallinckrodt Baker B.V. (Deventer, The Netherlands). Pyrrole (99 %), thiophene (99 %), and tetrabutylammonium perchlorate (98 %) were purchased from Sigma-Aldrich (Schnelldorf, Germany). Medical steel wires (Ni–Cr, ϕ = 750 μm) were purchased from B. Braun Surgical S.A. (Rubí, Barcelona, Spain). 

**Fig. 4:**
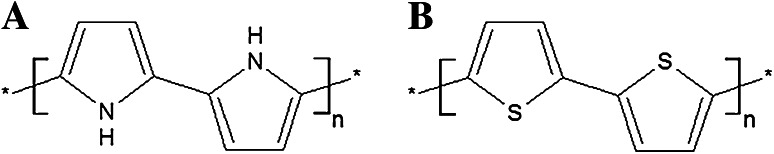
Chemical structures of PPy (**a**) and PTh (**b**) SPME fiber coatings

Polypyrrole (PPy) and polythiophene (PTh) coatings were synthesized by electropolymerization with a linear sweep voltammetry technique. We selected a potentiodynamic rather than potentiostatic technique for electropolymerization based on previous study [10]. The three-electrode cell was filled with electrolyte solution consisting of 100 mM pyrrole/thiophene (Fig. 4) and 250 mM tetrabutylammonium perchlorate in acetonitrile.

**Fig. 5:**
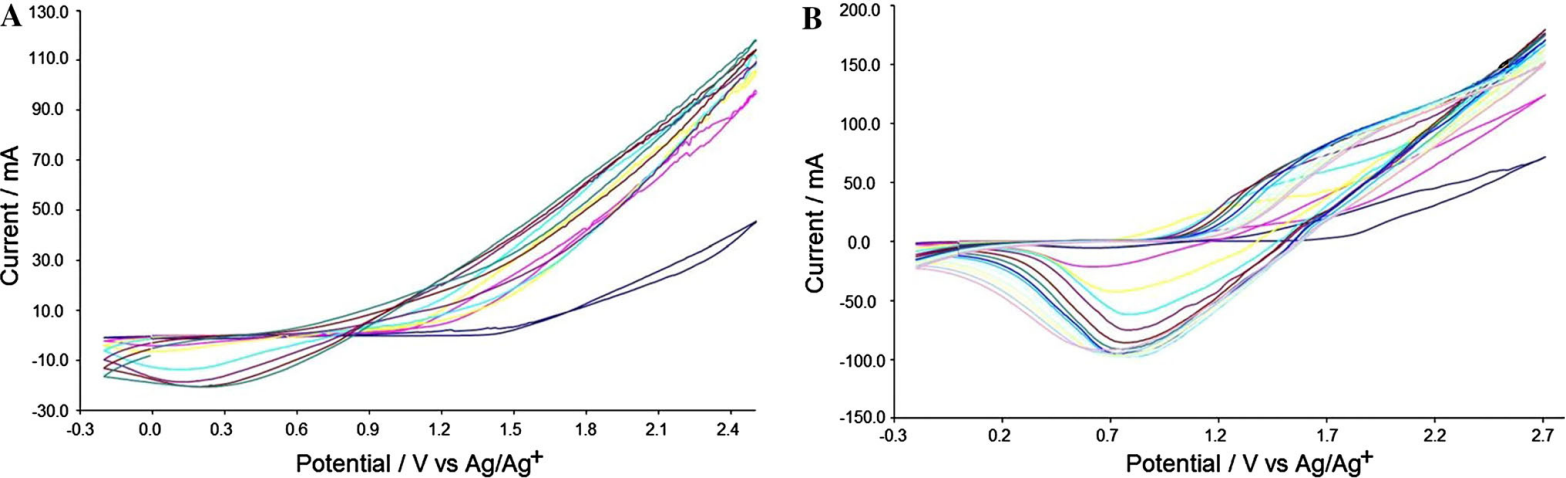
Electropolymerization of pyrrole (**a**) and thiophene (**b**)

Polymerization was performed using medical steel wires as working electrodes. An Ag/Ag^+^ electrode was applied as a reference electrode, with platinum net bent into a cylinder as a counterelectrode. For PPy, a potential range from −0.2 to +2.5 V and seven scans were applied (Fig. 5a), whereas for PTh a two-step process was used. First, the same conditions as for PPy were applied. Second, an additional 14 scans in the higher potential range from −0.2 to 2.7 V were used (Fig. 5b). The polarization speed was 50 mV s^−1^.

### Scanning electron microscopy

The morphologies of the polypyrrole and polythiophene fibers were investigated using a scanning electron microscope (LEO 1430VP; Carl Zeiss SMT, Oberkochen, Germany) coupled with a backscattered electron (BSE) detector.

### Small angle X-ray scattering

Small angle X-ray scattering (SAXS) data were collected using a NanoSTAR system (Bruker AXS GmbH, Karlsruhe, Germany) with pinhole collimation and a two-dimensional Hi STAR detector with resolution of 1,024 × 1,024 pixels, mounted on an X-ray tube with a copper anode and equipped with crossed Göbel focusing mirrors. Samples were mounted between two mica windows in sample holders with thickness of 1 or 2 mm. The sample-to-detector distance was 650 mm, and the exposure time for a single frame was 10,000 s. Each sample was measured three times, and the obtained data were averaged. The SAXS data were recorded within the scattering vector range of 0.15 nm^−1^ < *s* < 3.5 nm^−1^ (where *s* = 4*π*sin*θ*/*λ*, 2*θ* is the scattering angle, and *λ* is the X-ray wavelength). The recorded images were integrated using the spherical averaging method. The SAXS data were corrected for the detector response and normalized to the intensity of the incident beam, and the background scattering (empty holder) was subtracted using the SAXS_NT v4.1 program package (Bruker AXS GmbH, Karlsruhe, Germany).
